# Exploring the nexus between FDI inflows and economic growth: A sectoral level analysis

**DOI:** 10.1371/journal.pone.0301220

**Published:** 2024-05-17

**Authors:** Guo ai-jun, A. K. M. Mohsin, Sayed Farrukh Ahmed, Mst. Shumshunnahar, Arifur Rahman, Ebrahim Abbas Abdullah Abbas Amer, Hasanuzzaman Tushar

**Affiliations:** 1 School of Economics, Lanzhou University, Gansu, Lanzhou, China; 2 Faculty of Business & Entrepreneurship, Department of Business Administration, Daffodil International University, Daffodil Smart City, Ashulia, Dhaka; 3 Department of Public Administration, Comilla University, Cumilla, Bangladesh; 4 College of Business Administration, IUBAT University, Dhaka, Bangladesh; Royal Melbourne Institute of Technology, AUSTRALIA

## Abstract

This study investigates the relationship between Foreign Direct Investment (FDI) inflows and economic growth at sectoral levels in Bangladesh, employing a panel study framework. Utilizing sectoral-level panel data spanning six sectors from 2007–08 to 2018–19, the analysis is conducted using Panel Vector Error Correction Model (Panel VECM). Results from panel unit root tests confirm that all variables are integrated of order one *I (1)*, indicating stationarity. The Pedroni panel co-integration test further supports the presence of co-integration among the variables. Notably, the Panel VECM reveals evidence of a unidirectional causal relationship from Real Gross Domestic Product (RGDP) to Real Foreign Direct Investment (RFDI) across all six sectors of Bangladesh. The findings underscore the significance of formulating pragmatic policies and implementing them effectively to attract FDI across sectors, thereby contributing to the overall economic growth of Bangladesh.

## 1. Introduction

Foreign Direct Investment (FDI) is the investment from one country (home country) into another country (host country) in an attempt to ensure a substantial degree of influence or control on the enterprises of the host country [1]. Countries with ample capital resources constantly look for opportunities to enter into foreign markets to get maximum return from investment in host countries [2–4] with sustainable consumption. On the other hand, countries, suffering from capital shortages, are inclined to attract FDI to fill-up their saving-investment gap, increase knowledge as well as technological spillovers, and enrich their economic development as well as non-linear effects [5–7].

In addition to supply of capital in the host countries, FDI provides advanced technology and managerial know-how to the host economies, contributing to the host country’s development endeavor [8–12]. Moreover, some studies have documented that countries having superior growth rates are in an advantageous position to attract larger amounts of FDI [2,13–17]. Furthermore, countries with established financial structures, stable political conditions, bureaucratic efficiency, improved infrastructures, efficient human capital and stable economic situation can attract substantial amount of FDI [13,18].

Empirically, a good number of studies [19–28] have focused their attention on the relationship between FDI and host country’s economic growth at sectoral-level in a panel study framework. Some studies [19,21–27], using sectoral level data, have suggested that the FDI’s effect on economic growth differ across various sectors. This study is an attempt to fill the gap in the extant literature with a contribution in the area of the relationship between FDI and economic growth at sectoral level.

Since after independence, Bangladesh has attracted FDI in major sectors of economy including Agriculture and Fishing; Power; Pharmaceuticals and Chemicals; Gas and Petroleum; Textiles and Wearing; Fertilizer; Cement; Food Products; Leather Products; Trade and Commerce; Services; Transport, Storage and Communications; Construction etc. During the fiscal year (FY) 2020–21, major sectors of Bangladesh that attracted FDI inflows (Net) include Power (US$456.62 million), Textiles and Wearing (US$376.78 million), Food Products (US$307.31 million), Telecommunication (US$243.10 million), Banking (US$240.56 million), and Gas and Petroleum (US$150.09 million) which accounted for 18.2%, 15%, 12.3%, 9.7%, 9.6% and 6%, respectively of total FDI inflows (Net) of US$2507.31 million [29].

It is interesting to note that the economic studies on the relationship between total FDI and aggregate growth were predicated on the shaky premise that FDI in various sectors would have an equal influence on economic growth and would have homogeneous features [22]. It is not reasonable to expect FDI to have the same economic effects throughout an economy’s sectors. This is because each of these sectors has a distinct technological foundation, investment absorption capacity, and regulatory environment, among other factors [21]. The effect of FDI can therefore differ depending on sectoral specification for obvious reasons. Consequently, it would be beneficial to investigate the relationship between FDI and Bangladesh’s economic growth using sector-level data, as each sector has unique characteristics and, therefore, a significantly varied ability to generate influence from FDI.

While Foreign Direct Investment (FDI) has been drawn into various sectors of Bangladesh over an extended period, the specific effects of FDI on economic growth across different sectors remain largely unexplored. Notably, there is a scarcity of studies examining the relationship between FDI and economic growth using sectoral level data within the context of Bangladesh. Consequently, policymakers are confronted with the absence of a definitive answer regarding the extent of FDI’s impact on the economic growth of Bangladesh at the sectoral level. Although a solitary study [30] has addressed the effect of FDI on sectoral economic growth in Bangladesh, utilizing data from 1995–2005, there remains an opportunity to conduct research using the latest sectoral data to glean fresh insights into the sector-specific effects of FDI on Bangladesh’s economic growth.

This study aims to bridge this gap by investigating the relationship between FDI and economic growth in Bangladesh using sectoral level data. The unique focus on this individual, country-specific study, particularly at the sectoral level, is expected to make a significant contribution to empirical research on the relationship between FDI and economic growth using sectoral level panel data. The study is motivated by the potential findings regarding the relationship between FDI and economic growth at the sectoral level, which can contribute to the growing literature in this area and provide valuable insights for policymakers in formulating targeted policies to attract FDI into specific sectors.

The paper is structured as follows: Section 2 reviews relevant empirical literature. Section 3 outlines the data and methodology employed. Section 4 presents the results and provides discussion. Finally, Section 5 concludes the study with pertinent policy implications.

## 2. Literature review

Several empirical studies [19–28,31] have delved into the relationship between Foreign Direct Investment (FDI) and economic growth at the sectoral level. Some of these studies [19,21–27], utilizing sectoral level data, have highlighted variations in the impact of FDI on economic growth across different sectors. Additionally, certain studies [20,25–27] have pointed out challenges related to the reliability and availability of sectoral level data, which can hinder empirical research in this area.

For instance, [32] employed the 2SLS approach to analyze the impact of sectoral FDI on economic growth across 85 developing countries from 1996 to 2019. Their findings underscored the significant role played by sectoral FDI inflows in driving economic growth in these countries. Specifically, they found that while services and manufacturing FDI have limited growth-promoting effects in low-income nations, industry and agriculture FDI are more impactful. Moreover, FDI inflows were found to stimulate economic growth across all sectors in high-income nations, except for services.

[33] used panel data estimate methodologies and data from 2011 to 2019 to study the effects of FDI sectors on the economic growth of 10 ex-socialist Asian and European nations. The study found that FDI inflows into the industrial sector significantly affect growth. Interestingly, not all FDI inflows into the manufacturing sector boost economic growth, according to the empirical study conducted at the subsector level. In particular, the findings demonstrated that, out of 13 subsectors, only 6 subsectors had statistically significant and favorable effects on growth from FDI inflows.

In the case of an emerging economy such as India, [34] investigated, using data from 1995 to 2016, how sector-wise FDI inflows can influence the growth of respective sectors. As per the VECM findings, inward FDI did not contribute to the growth in agricultural output. Nevertheless, a reverse causal relationship is observed, whereby more FDI in the agricultural sector is drawn to agricultural output. FDI inflow is observed to have a favorable impact on the manufacturing sector’s output. The study also confirmed a bidirectional causal relationship between FDI and growth in the service sector both in long run and short run.

[35] used sector-specific data from 2007 to 2016 to investigate the sectoral analysis of FDI on Nepal’s economic growth. The results suggested that FDI in the agriculture, tourism, and industry sectors have positive influence on Nepal’s economic growth over the given period.

In a recent study, employed Generalized Methods of Moments (GMM) estimation technique to examine the impacts of sectoral FDI on the economic growth of Egypt by using panel data of 26 Egyptian governorates over the period 1992–2007. The study found no evidence of significant effect of manufacturing FDI on the economic growth of the selected Egyptian governorates. The authors suggested that the Egyptian policymakers should focus on improvement of investment infrastructure and financial reforms to attract more FDI in various sectors.

In addition to these studies, research by [36] delved into the asymmetric effects of FDI on tourism demand in China over the period 1982–2017. Their study employed advanced methodologies including non-linear autoregressive distributed lag analysis and identified structural breaks using the Bai-Perron test. The results unveiled an asymmetric association between FDI and tourism demand, with declines in FDI having a more pronounced impact on tourism demand compared to increases. This research provides crucial insights for policymakers managing FDI in the tourism sector, emphasizing the need for nuanced strategies to navigate the fluctuations in FDI and their implications for tourism demand.

The study of [21] investigated the relationship between FDI and sectoral growth of Indian economy by using data of seven sectors (automobiles, telecom, services, metallurgy, chemical, pharmaceuticals and drugs, tourism) over the period from 2001 to 2014. Empirical findings of the study indicated that FDI exerts no significant effect on gross output of the entire sectors chosen, whereas gross output has positive and significant effect on FDI for the entire sectors chosen. With respect to the panel Granger causality test, the study revealed the evidence of bidirectional causal relationship between FDI and gross output. The authors further suggested that the Indian policymakers should focus on the development of financial sector, macroeconomic stability, and relaxation of the regulations for attracting higher FDI inflows into India.

[22] investigated the sector-specific impact of FDI on economic growth for Turkey by using panel data of 10 sectors between 2000 and 2009. The study concluded that there is long-run cointegrating relationship between FDI and GDP in Turkey and there exists unidirectional causality running from FDI to GDP which means that FDI has planted in first period, and then, GDP has exhibited an improved growth rate in second period. At the sectoral level, FDI facilitates growth rate of Turkey most in the manufacturing, power, gas and water, electricity, wholesale, and retail trade sectors.

[23] applied panel cointegration framework to investigate the empirical relationship between FDI and output at sectoral levels for Pakistan by using panel data of 23 industries for a period of 1981–2008. Their results found one-way causality running from GDP to FDI in long-run and two-way causality between FDI and GDP in short-run. Moreover, the study also suggested that the impact of FDI on growth differs broadly across diverse sectors. They indicated that in the primary and service sectors, growth is caused by FDI, while in the manufacturing sector, it is growth that stimulates FDI.

[24] applied random effect model and weighted least squares (WLS) method to examine the heterogeneous effects of sector-level inflows of FDI on the economic growth of host country by using data of 12 Asian countries over the years from 1987 to 1997. The study showed that FDI in various sectors does have diverse effects on the economic growth of host country. Specifically, the study revealed that manufacturing FDI has significant and positive impact on the economic growth of host countries chosen for study, whereas non-manufacturing FDI does not play important role in enhancing growth. The author suggested to adopt favorable investment-friendly policies for attracting substantial amount of FDI in specific sectors.

The study of [30] endeavored to examine the effect of FDI on the sectoral economic growth of Bangladesh by considering sectoral data (industry, agriculture, and service) from 1995 to 2005. The study found correlation between FDI in the service sector with service sector growth, whereas, in case of FDI in the industrial sector and FDI in the agricultural sector, the study found no correlation.

[26] investigated the role of sectoral composition of FDI inflows on economic growth by using data of 33 countries over the period from 1990 to 2002. The findings of the study confirmed that the composition of FDI inflows influence the economic growth of host country. The study revealed growth effects when FDI in manufacturing sector captures a significant portion, but negative growth effects when FDI in primary or service sector is very high.

In their study, [27] applied fixed effect model to explore the effects of FDI on the economic growth at different sectoral levels of Indonesia by using data of 12 sectors over the period from 1997 to 2006. The study revealed that FDI appears to have positive impact on economic growth at aggregate level of Indonesia, while the impacts of FDI on growth at sectoral level differ across sectors. In addition, the authors emphasized on appropriate sectoral composition of FDI in host country and further suggested to formulate effective policies for ensuring maximum benefits from inflows of FDI.

In a study, [19] empirically explored the impact of FDI on economic growth by considering sectoral data of 47 countries over the period 1981–1999. The study revealed that the effect of FDI differ significantly across sectors (primary, services, manufacturing) for the countries chosen for the study. In addition, FDI in manufacturing sector tends to have positive impact on growth, evidence from primary sector is negative one, whereas FDI in services sector tends to have no significant contribution to economic growth.

The existing literature utilizing panel data has yielded diverse findings concerning the effects of Foreign Direct Investment (FDI) on economic growth across various sectors and countries. While some sectors experience growth due to FDI inflows, others witness a reverse causality where growth stimulates FDI. Conversely, certain studies have found no discernible causal relationship between FDI and economic growth in specific sectors. Moreover, conflicting results have been observed, with some studies reporting positive effects of FDI on growth while others highlight negative impacts across multiple sectors.

Despite the extensive research in this field, there is a notable scarcity of empirical studies investigating the relationship between Foreign Direct Investment (FDI) and economic growth at the sectoral level, especially in developing countries like Bangladesh. Despite Bangladesh’s consistent attraction of FDI across various sectors, policymakers are still uncertain about the presence and characteristics of this relationship at the sectoral level. By analyzing this relationship using sectoral-level data, we can not only clarify how FDI impacts Bangladesh’s economic growth in specific sectors but also assist policymakers in crafting precise FDI policies tailored to each sector’s needs.

Therefore, it is imperative to explore the relationship between FDI and economic growth in Bangladesh using sectoral level data. Such research has the potential to make a distinctive contribution not only to the literature on Bangladesh but also to the global understanding of FDI’s impact on economic growth at the sectoral level. The findings can provide valuable insights into the real impact of FDI on economic growth and inform policymakers in designing effective policies to attract FDI to specific sectors.

## 3. Data and methodology

### 3.1. Data

In the study, sectoral level panel data of six different sectors (Agriculture and Fishing; Manufacturing; Power; Construction; Transport, Storage and Communication; Financial Intermediations) of Bangladesh over the period from 2007–08 to 2018–19 have been used. The selection of six sectors and time periods of twelve years is driven by data availability on the relevant variables. Due to the unavailability of data on the relevant variables of the mentioned time period, data of some sectors (such as gas and petroleum, trading, services) could not be considered for the study. In the study, the period of twelve years may not be a problem as the period of twelve years or less had been used in several panel studies [22,27,28,37,38].

Over the study period from 2007–08 to 2018–19, FDI net inflows brought by six sectors was about 86%, on average [29]; whereas six sectors contributed about 65% to real GDP (constant 2005–06 BDT), on average [39]. The study is limited to the bivariate relationship between FDI and economic growth in a panel study framework in the context of Bangladesh. In the relevant literatures of panel study, this restriction is fairly common. In similar types of study, [25,38,40] had used the bivariate approach. The detailed description of the variables used in the study is provided in Table 1 below.

**Table 1:** Description of the variables.

Variable^a^	Description
RGDP	RGDP stands for Real Gross Domestic Product (constant 2005–06 BDT). In this study, the variable is used following [25]. Data of the variable has been obtained from [39].
RFDI	RFDI stands for Real Foreign Direct Investment net inflows. In this study, the variable is used following [19,26]. Data of FDI net inflows (current million US$) has initially been transformed in local currency (BDT) and then converted to real values by dividing the calculated current values by the GDP deflator (2005–06 = 1), using 2005–06 as the base year following [38]. Data of the variable has been obtained from [29]. Foreign Direct Investment (FDI) is the investment from one country (home country) into another country (host country) in order to ensure significant degree of influence or control on the enterprises of the host country [1].

Note: ^a^All the variables have been measured in real terms (constant 2005–06 BDT) following [38,41–43].

In the study, all the variables have been transformed in logarithmic forms for avoiding the scaling problem. It may help to avoid the sharpness as well as the variations in the data so that coefficients are not affected by the extreme values or outliers [44].

### 3.2. Methodology

The study regarding the relationships between FDI inflows and economic growth of Bangladesh at sectoral levels in a panel study framework follows the three-step procedure as suggested by [25,42]. First, panel unit root of each variable used in the study has been tested. Second, upon getting the confirmation that the studied variables are integrated order, *I(1)*, panel co-integration test suggested by [45] has been used to test the long-run co-integration relationship between the studied variables. Finally, given the existence of co-integration, the Panel Vector Error Correction Model (VECM) has been applied to investigate the causal relationship between the variables.

Numerous panel unit root tests proposed by [46–49] have been applied for identifying the stationary properties of the panel data. [48] indicated unit root tests for dynamic heterogeneous panels based on the mean of specific unit root statistics. The authors proposed the IPS test suggesting a standardized *t*-bar test statistic which can be stated as follows:

$$t_{IPS}=\frac{\sqrt{N}\left(\bar{t}-\frac{1}{N}\sum_{i=1}^{N}E\left[t_{iT}|\rho_{i}=0\right]\right)}{\sqrt{\frac{1}{N}\sum_{i=1}^{N}\mathrm{var}\left[t_{iT}|\rho_{i}=0\right]}}\Rightarrow N(0,1)$$ (1)

where values of *E*[*t*_*iT*_|*ρ_i_* = 0] and var[*t*_*iT*_|*ρ_i_* = 0] have been found from the outcomes of Monte Carlo simulations.

[47] takes into account pooling cross-section time series data with a view to testing the unit root hypothesis. The adjusted *t*-statistic proposed by [47] is as follows:

$$t_{\delta }^{*}=\frac{t_{\delta }-N\tilde{T}{\hat{S}}_{N}{\hat{\sigma }}_{\tilde{\varepsilon }}^{-2}STD(\hat{\delta })\mu_{m\tilde{T}}^{*}}{\sigma_{m\tilde{T}}^{*}}$$ (2)

where $\mu_{m\tilde{T}}^{*}$ denotes the mean adjustment and $\sigma_{m\tilde{T}}^{*}$ denotes the standard deviation adjustment. In addition, the adjusted t-statistic $t_{\delta }^{*}$ follows the standard normal distribution, asymptotically.

[49] suggested a Fisher-type test combining the *p*-values from unit root tests for every cross-section (*i)* with a view to testing the unit root in the panel data which can be stated as follows:

$$p^{\lambda }=-2\sum_{i=1}^{N}\log_{e}\pi_{i}$$ (3)

where the significance levels *π*_*i*_(*i* = 1,2, 3…, *N*) have been considered independent uniform (0, 1) variables.

[46] suggests a test statistic which does not use biased adjustment and whose power is significantly greater than the adjusted *t*-statistic suggested by [47] or the *t*-statistic of IPS test suggested by [48]. The statistic of Breitung is as follows:

$$\lambda_{B}=\frac{\sum_{i=1}^{N}\sigma_{1}^{-2}y_{i}^{{*}^{\prime }}x_{i}^{{*}^{\prime }}}{\sqrt{\sum_{i=1}^{N}\sigma_{1}^{-2}x_{i}^{{*}^{\prime }}A^{\prime }AX_{i}^{*}}}$$ (4)

which has a standard normal distribution as (*N*, *T* →∞)_*seq*_

Upon getting the confirmation that all of the variables, based on the outcomes of panel unit root tests, are integrated order, *I(1)*, panel co-integration test proposed by [45] has been used to detect the long-run co-integration relationship between the studied variables.

[45] suggests the following panel regression:

$$y_{i,t}=\alpha_{i}+\delta_{i}t+\beta_{1i}x_{1i,t}+\beta_{2i}x_{2i,t}+\ldots ‥+\beta_{mi}x_{mi,t}+e_{i,t}$$ (5)

fort=1,‥‥,T;i=1,….,N;m=1,……‥,M

where *T* denotes the number of observations over time, *N* denotes the number of individual members in the panel and *M* denotes the number of regression variables. The parameter (*α*_*i*_) denotes the member-specific intercept that differs across individual members. The slope coefficients (*β*_1*i*_, *β*_2*i*_, ……‥, *β*_*mi*_) differ across individual members in the panel. The term *δ*_*i*_*t* represents deterministic time trends that are said to be specific to individual members of the panel.

[45] proposes seven statistics that test the null hypothesis of no co-integration against co-integration in the panel data. Of these seven statistics, four are called panel co-integration statistics (within dimension-based statistics) and three are referred to as group-mean panel co-integration statistics (between dimension-based statistics).

Given the existence of co-integration between the studied variables, the panel VECM has been applied to examine the causal relationship between the variables which not only identifies the sources of causation but also differentiates between the long-run and the short-run relationship in the series. Panel VECM fails to provide individual sector test output for which sector-wise result cannot be shown [25,42].

In the present study, the panel VECM has been used which has numerous benefits. It allows for the interpretation of both long-term and short-term equations. Using VECM, the first differenced variables and error correction term could be determined. Coefficient estimates in the VAR that results from the VECM representation are more accurate [25,42].

Following [25,41,42], a bivariate panel VECM for investigating the causality between RFDI and economic growth (RGDP) may be stated as below:

$$\Delta LRGDP_{it}=c_{1i}+\sum_{i=1}^{k}\alpha_{1ik\;}\Delta LRGDP_{it-k}+\sum_{i=1}^{k}\beta_{1ik\;}\Delta LRFDI_{it-k}+\varphi_{1i}ECT_{t-1}+\varepsilon_{it}$$ (6)

$$\Delta LRFDI_{it}=c_{2i}+\sum_{i=1}^{k}\alpha_{2ik\;}\Delta LRGDP_{it-k}+\sum_{i=1}^{k}\beta_{2ik}\;\Delta LRFDI_{it-k}+\varphi_{2i}ECT_{t-1}+\epsilon_{it}$$ (7)

where Δ denotes the first difference operator; *ECT*_*t*-1_ denotes the lagged error correction term; *k* denotes the lag length; *ε*_*it*_ and *ϵ*_*it*_ denote the serially uncorrelated error terms.

In Eqs (6) and (7), two coefficients *φ*_1*i*_ and *φ*_2*i*_ denote the speeds of adjustment along the long-run equilibrium path. Failing to reject H_0_: *φ*_1*i*_ = 0 for all *i* (*i* = 1, 2, …, 6), indicates that RFDI does not Granger cause RGDP for any of the sectors included in the panel in the long run. On the other hand, failing to reject H_0_: *φ*_2*i*_ = 0 for all *i* (*i* = 1, 2, …, 6), means that RGDP does not Granger cause RFDI for any of the sectors included in the panel in the long run. Besides, failing to reject H_0_: *β*_1*ik*_ = 0 for all *i* (*i* = 1, 2, …, 6) and *k* (*k* = 1,2,…‥,*k*) suggests that RFDI does not Granger cause RGDP for any of the sectors included in the panel in the short run. Moreover, failing to reject H_0_: *α*_2*ik*_ = 0 for all *i* (*i* = 1, 2, …, 6) and *k* (*k* = 1,2,…‥,*k*) indicates that RGDP does not Granger cause RFDI for any of the sectors included in the panel in the short run.

## 4. Results and discussion

Table 2 presents the descriptive statistics of the variables. It is apparent that average RGDP is US$9151.67 million. It ranges from US$790.84 million to US$27316.69 million. On the other hand, average RFDI is US$131.19 million. It ranges from US$0.14 million to US$472.73 million.

**Table 2:** Descriptive statistics of the variables.

Variable	Obs.	Mean	Max.	Min.	Std. Dev.
RGDP	72	9151.67	27316.69	790.84	6571.61
RFDI	72	131.19	472.73	0.14	141.96

Source: Authors’ calculation based on [29] and Bangladesh Economic Review (2021). Variables are in Million US$.

The standard deviations indicate higher variation in the data across sector and over time of the variable RGDP compared to RFDI. Table 3 shows descriptive statistics (within and between variations) of the variables of the study.

**Table 3:** Descriptive statistics (within and between variations) of the variables.

Variable		Mean	Std. Dev.	Min.	Max.	Obs.
RGDP	overall	9151.67	6571.61	790.84	27316.69	N = 72
	between		6772.60	1144.81	18952.16	n = 6
	within		2077.20	3783.67	17516.2	T = 12
RFDI	overall	131.19	141.96	0.14	472.73	N = 72
	between		127.56	4.89	339.86	n = 6
	within		79.77	-58.62	397.84	T = 12

Source: Authors’ calculation based on [29,39]. Variables are in Million US$.

From Table 3, it can be concluded that average RGDP for each sector varies between US$1144.81 million and US$18,952.16 million. The calculated standard deviation indicates that the variation in RGDP across sectors is US$6772.60 million and the variation in RGDP within a sector over time is US$2077.20 million. On the other hand, average RFDI for each sector varies between US$4.89 million and US$339.86 million. The calculated standard deviation shows that the variation in RFDI across sectors is US$127.56 million and the variation in RFDI within a sector over time is US$79.77 million. The results of various panel unit root tests of LRFDI and LRGDP are shown in Table 4 below.

**Table 4:** Panel unit root tests.

Test	LRFDI		LRGDP	
	Level	1^st^ diff.	Level	1^st^ diff.
[47] Levin, Lin & Chu	-4.77***	-6.69***	-4.12***	-6.16***
[48] Im, Pesaran and Shin	-0.58	-1.02	-0.14	-0.58
[46] Breitung t-stat	-0.66	-1.83**	1.59	-0.90
[50] ADF-Fisher Chi-square	18.01	24.77**	16.31	19.05*
[51] PP-Fisher Chi-square	14.79	33.05***	20.48*	21.73**

Notes: ***Significant at 1 percent level; **Significant at 5 percent level; *Significant at 10 percent level.

Table 4 shows that most of panel unit root tests fail to reject the null hypothesis of unit root at levels, meaning that LRFDI and LRGDP are non-stationary at levels, but the results of panel unit root tests in the first difference suggest that all the variables are stationary after the first difference because most of these tests reject the null hypothesis of unit root in the first difference. That is to say, the variables are integrated of order one, *I(1)*.

With the confirmation that all of the variables, based on the results of panel unit root tests, are integrated order, *I(1)*, panel co-integration test as proposed by has been applied to check the long-run co-integration relationship between the variables.

Before applying the Pedroni panel co-integration test, the optimal lag length has to be specified.

As is apparent from Table 5, the optimal lag of two has been selected on the basis of AIC, SC, LR, FPE and HQ.

**Table 5:** Lag length selection.

Lag	LR	FPE	AIC	SC	HQ
0	NA	2.590417	6.627524	6.720937	6.657407
1	255.2970	0.000265	-2.561254	-2.281014	-2.471603
2	18.63596*	0.000165*	-3.040025*	-2.572959*	-2.890607*
3	1.455455	0.000205	-2.836639	-2.182747	-2.627453
4	5.208241	0.000213	-2.817984	-1.977265	-2.549031
5	2.183209	0.000256	-2.666223	-1.638678	-2.337503

Source: Authors’ own Calculation based on Data. Note:

* indicates lag order selected by the criterion.

Table 6 shows the results of Pedroni panel co-integration test. All Pedroni statistics, except Panel v-statistic, Panel rho-statistic, and Group rho-statistic, reject the null of no co-integration, thereby indicating the co-integration between LRFDI and LRGDP.

**Table 6:** Pedroni panel co-integration test.

Statistics	Statistic	Prob.
Panel v-statistic	-1.89	0.970
Panel rho-statistic	0.81	0.792
Panel PP-statistic	-2.22^**^	0.012
Panel ADF-statistic	-3.35^***^	0.000
Group rho-statistic	2.04	0.979
Group PP-statistic	-1.73^**^	0.041
Group ADF-statistic	-2.59^***^	0.004

Notes: ***Significant at 1 percent level; **Significant at 5 percent level.

Thus, the results of Pedroni panel co-integration test support the co-integration between LRFDI and LRGDP as majority of the statistics suggest the rejection of null of no co-integration. With the affirmation that LRFDI and LRGDP are cointegrated based on the results of Pedroni panel co-integration test, the panel VECM can be applied for identifying the sources of causation as well as distinguishing between the long-run and the short-run relationship of the series. As mentioned in the methodology part, Panel VECM fails to provide individual sector test output for which sector-wise result cannot be shown [25,42].

Table 7 shows the result of panel VECM for RGDP equation (Eq 6). From Table 7, it is apparent that the coefficient of the ECT (ECT_t-1_), is negative (-0.00016) but not statistically significant, thereby indicating no long-run causality running from RFDI to RGDP. The possible reason may be that FDI in Bangladesh, particularly at the sectoral level, could not contribute to the sectoral economic growth. The outcome is consistent with the results of similar types of previous panel studies [20,21,23].

**Table 7:** Result of panel VECM for RGDP equation (Eq 6).

Dependent Variable	Sources of Causation			Short-run Relationship	Long-run Relationship
	Short-run		Long-run		
	ΔLRFDI	ΔLRGDP	ECT_t-1_		
ΔLRGDP	0.0995 (0.9515)	-	-0.00016 (0.6535)	No	No
Diagnostic tests			Result	Decision	
Residual Cross-section Dependence Test (Pesaran CD)			1.119 (0.2629)	There is no cross-section dependence (correlation) in residuals	
Jarque-Bera test for normality			15.94^***^ (0.000)	Residuals are not normally distributed	

Notes: *** indicates 1% level of significance; corresponding p-values are in parentheses.

Moreover, the null hypothesis that there is no short-run causality is not rejected indicating that there is no evidence of short-run causal relation running from RFDI to RGDP when considering the entire panel of 6 sectors. The finding is consistent with the results of [21].

Bottom panel of Table 7 shows the results of different diagnostic tests. Panel data models could show cross-sectional dependence in the errors resulting from the common shocks as well as unobserved components [52]. Ignoring cross-sectional dependence in estimation may result in invalid test statistics and estimator efficiency loss [50]. The Residual Cross-section Dependence Test indicates no cross-section dependence in residuals. The residuals are not found normally distributed as suggested by the Jarque-Bera (JB) test for normality.

Table 8 shows the result of panel VECM for RFDI equation (Eq 7). From Table 8, it is evident that the coefficient of the ECT (ECT_t-1_) is negative (-0.201) and statistically significant at 1 percent level, indicating the long-run equilibrium relationship between RFDI and RGDP. More specifically, it can be said that there is the evidence of long-run causality from RGDP to RFDI. It means that 20.1 percent of disequilibrium in the long-run relationship is corrected each period into its equilibrium or the whole system is getting back to long-run equilibrium at the speed of 20.1 percent annually or it requires about 4.98 years to reach the long-run equilibrium.

**Table 8:** Result of panel VECM for RFDI equation (Eq 7).

Dependent Variable	Sources of Causation			Short-run Relationship	Long-run Relationship
	Short-run		Long-run		
	ΔLRFDI	ΔLRGDP	ECT_t-1_		
ΔLRFDI	-	6.202^**^ (0.045)	-0.201^***^ (0.0005)	RGDP causes RFDI	Yes
Diagnostic tests			Result	Decision	
Residual Cross-section Dependence Test (Pesaran CD)			-0.495 (0.6199)	There is no cross-section dependence (correlation) in residuals	
Jarque-Bera test for normality			1.104 (0.575)	Residuals are normally distributed	

Notes: *** and **indicate 1% and 5% level of significance, respectively; corresponding p-values are in parentheses.

Moreover, the null hypothesis of no short-run causality is rejected at 5 percent level of significance indicating the short-run causal relation running from RGDP to RFDI when considering the entire panel of 6 sectors. It may happen that rapid economic growth requires more investments including FDI for further development. The host country’s better economic performance may create higher opportunities for making profits from investment which may encourage foreign investors to invest more in expectation of greater profit. The outcome is consistent with the results of [21]. Bottom panel of Table 8 shows the results of different diagnostic tests. The Residual Cross-section Dependence Test finds no cross-section dependence in residuals. The residuals are found to be normally distributed as indicated by the JB test for normality.

Thus, the research results recommend the evidence of unidirectional causal relation running from RGDP to RFDI when considering the entire panel of 6 sectors of Bangladesh. The probable reason may be that perhaps rapid economic growth may give favorable signal to the foreign investors about the country’s economic progress, thus encourage them to invest more in expectation of higher expected profits. This outcome is similar to the ones of [21]. Policymakers may need to devise and implement effective policies for ensuring sectoral economic growth with the help of FDI allocated in specific sectors. Policymakers not only have to set pragmatic policies but also implement those policies efficiently for inviting FDI in the sectors, keeping in mind the importance of the sectors in the economy. The probable effects of FDI projects on specific sectors (i.e., expected benefits from these FDI projects) may need to be evaluated before allowing FDI into those sectors.

## 5. Conclusion, implications, limitations and future research direction

The key contribution of the study is the examination of the relationship between FDI inflows and economic growth of Bangladesh at sectoral levels in a panel study framework by using sectoral level panel data of six different sectors (Agriculture and Fishing; Manufacturing; Power; Construction; Transport, Storage and Communication; Financial Intermediations) of Bangladesh over the period from 2007–08 to 2018–19. Firstly, various panel unit root tests have been performed and the results indicate that all the variables (LRFDI and LRGDP) are integrated of order one, *I(1)*. Secondly, the results of Pedroni panel co-integration test support the existence of co-integration between LRFDI and LRGDP. Finally, with the affirmation that LRFDI and LRGDP are cointegrated based on the results of Pedroni panel co-integration test, the panel VECM has been applied which suggests the evidence of long-run causality from RGDP to RFDI and unidirectional short-run causal relation running from RGDP to RFDI when considering the entire panel of 6 sectors.

The contribution of the study and empirical findings lead to significant policy implications for RFDI and economic growth (RGDP) of Bangladesh at sectoral levels which is consistent to the prior study [30]. The evidence of unidirectional causal relation running from RGDP to RFDI indicates the fact that perhaps rapid economic growth may give favorable signal to the foreign investors about the country’s economic progress, thus encourage them to invest more in expectation of higher expected profits. It may be worthwhile for the policymakers to evaluate the pros and cons of each of the FDI projects and its probable impact on specific sectors (i.e., expected benefits from these FDI projects) before allowing FDI into those sectors [9,12]. Special attention may need to be given to the improvement of business environment (i.e., ease of doing business index), implementations of necessary sectoral reforms, specialization in production process, good governance, and human-capital development along with attracting foreign investment for ensuring economic development at sectoral levels.

However, the study suffers from some limitations. The selection of six sectors and time periods of twelve years is driven by data availability on relevant variables. Inclusion of more sectors with extended time periods may bring diversified outcomes and make the study more exhaustive. Besides, analysis of sector-wise disaggregation including sector-specific causality test, subject to the availability of data, may be conducted in further study to understand the nature of causal links between FDI and economic growth across various sectors. Future research may focus on the big data analytics, mixed methodology analysis and cross-country analysis in various time periods.

## Supporting information

S1 File (XLSX)
